# Accelerated Metabolomic Aging and Its Association with Social Determinants of Health in Multiple Sclerosis

**DOI:** 10.1101/2025.01.29.25321260

**Published:** 2025-03-24

**Authors:** Fatemeh Siavoshi, Rezvan Noroozi, Gina Chang, Vinicius A. Schoeps, Matthew D. Smith, Farren B.S. Briggs, Jennifer S. Graves, Emmanuelle Waubant, Ellen M. Mowry, Peter A. Calabresi, Pavan Bhargava, Kathryn C Fitzgerald

**Affiliations:** 1 Department of Neurology, Johns Hopkins University School of Medicine, Baltimore, MD, USA; 2 Department of Neurology, Children’s Hospital of Philadelphia, Philadelphia, PA, USA; 3 Department of Neurology, University of California, San Francisco, San Francisco, CA, USA; 4 Department of Epidemiology, Johns Hopkins University School of Public Health, Baltimore, MD, USA; 5 Department of Public Health Sciences, University of Miami Miller School of Medicine, Miami, FL, USA; 6 Department of Neurosciences, University of California, San Diego, San Diego, CA, USA.

## Abstract

**Objectives:**

Biological age may better capture differences in disease course among people with multiple sclerosis (PwMS) of identical chronological age. We investigated biological age acceleration through metabolomic age (mAge) in PwMS and its association with social determinants of health (SDoH) measured by the area deprivation index (ADI).

**Methods:**

mAge was calculated for three cohorts: 323 PwMS and 66 healthy controls (HCs); 102 HCs and 72 DMT-naïve PwMS; and 64 HCs and 67 pediatric-onset MS/clinically isolated syndrome patients, using an aging clock derived from 11,977 healthy adults. mAge acceleration, the difference between mAge and chronological age, was compared between groups using generalized linear and mixed-effects models, and its association with ADI was assessed via linear regression.

**Results:**

Cross-sectionally, PwMS had higher age acceleration than HCs: 9.77 years in adult PwMS (95% CI:6.57–12.97, p=5.3e-09), 4.90 years in adult DMT-naïve PwMS (95% CI:0.85–9.01, p=0.02), and 6.98 years (95% CI:1.58–12.39, p=0.01) in pediatric-onset PwMS. Longitudinally, PwMS aged 1.19 mAge years per chronological year (95% CI:0.18, 2.20; p=0.02), faster than HCs. In PwMS, a 10-percentile increase in ADI was associated with a 0.63-year (95% CI:0.10–1.18; p=0.02) increase in age acceleration.

**Discussion:**

We demonstrated accelerated mAge in adult and pediatric-onset PwMS and its association with social disadvantage.

## Introduction

Chronological age (cAge), determined by birth date, is associated with multiple sclerosis (MS) progression and therapeutic response^1^. However, disease progression varies among people with MS (PwMS) of the same cAge, therefore, biological age (bAge), reflecting physiological aging and cellular health, may capture these differences more accurately.

Social determinants of health (SDoH), non-medical factors like living conditions, shape biological differences and influence physical and mental health outcomes, including those in PwMS^2,3^. In the general population, unfavorable SDoH are linked to earlier onset and greater severity of age-related diseases, suggesting they may accelerate biological aging^4^. This accelerated aging could also mediate the impact of SDoH on MS progression, highlighting the importance of exploring this relationship in MS.

This study compares bAge, estimated through metabolomic profiles, in PwMS and healthy controls (HCs) and examines its association with SDoH in PwMS. While bAge is commonly assessed using biomarkers like telomere length or clinical laboratory values, metabolomics provides additional insight by reflecting downstream products of biological processes influenced by genetic and environmental exposures relevant to SDoH^5,6^.

## Methods

### Study participants

Data were pooled from Johns Hopkins MS Precision Medicine Center of Excellence (JHU; including PwMS and HCs), University of Miami Miller School of Medicine (UMMSM; including HCs and disease-modifying therapy (DMT)-naïve PwMS [<5 years from symptom onset and ≤2 years from diagnosis]), and pediatric MS Center at the University of California, San Francisco (UCSF; including HCs and pediatric-onset MS/clinically isolated syndrome [POMS/CIS] patients within 4 years of symptom onset).

### Measure of social determinants of health

We used the national area deprivation index (ADI) as the primary SDoH indicator linked to health outcomes in the general population and PwMS. The ADI incorporates 17 socioeconomic status (SES) measures ranging from 1–100 (least to most disadvantaged)^2,7,8^. ADI analyses were limited to PwMS in the JHU cohort, as only these participants had the necessary geocoded data.

### Metabolomic measurements

Blood samples were processed within 3 hours of collection, stored at −80°C, and analyzed using an untargeted metabolomic platform by Metabolon Inc. (Durham, NC) as detailed previously^9^. Derivatized samples were analyzed using gas chromatography-mass spectrometry or liquid chromatography-tandem mass spectrometry. Mass spectra were compared to a reference library for metabolite identification, and relative abundances were calculated from the area under the curve. Profiling was conducted in five batches for JHU, two for UCSF, and in a single batch for the UMMSM cohort.

### Statistical analysis

#### Preprocessing and quality control

Metabolites with >20% missing values per batch were excluded, and the remaining missing values were imputed using k-nearest neighbors (k=10) and log-transformed, yielding 332, 646, and 959 metabolites in JHU, UCSF, and UMMSM, respectively. Within-site batch effects were harmonized using ComBat^10^. Finally, data were winsorized at the 99^th^ percentile, with JHU and UCSF converted to Z-scores and UMMSM scaled to a median of one.

#### Metabolomic age calculation

Metabolomic age (mAge) was calculated using a model developed based on the 11,977 healthy adult individuals from the INTERVAL study, profiling performed by Metabolon Inc^11^. The mAge model had two versions: Model A (including 678 endogenous and 148 xenobiotics) and Model B (678 endogenous). mAge was calculated by applying the intercept, sex coefficient, and the sum of metabolites obtained by multiplying each metabolite’s level by its corresponding coefficient for matching metabolites—332 in the JHU and 563 in the UCSF, and 731 in the UMMSM dataset—from Model A, as this model with both endogenous and xenobiotics, accounted for more environmental exposures. mAge values outside the range of 0–100 were excluded from downstream analysis (n=7 for the UMMSM cohort).

#### Descriptive and analytical models

Descriptive analyses summarized categorical variables as frequencies and continuous variables as means or medians. The primary effect was age acceleration, defined as the difference between mAge and cAge. For cross-sectional comparisons between PwMS and HCs in the UMMSM and UCSF cohorts (one sample per individual), generalized linear models were used. In the JHU cohort, with multiple samples for 195 individuals, a linear mixed-effects (LME) model was applied to account for within-person correlations. Longitudinally, in JHU participants with follow-up exceeding one year, an LME model was employed to evaluate whether PwMS exhibited faster biological aging compared to HCs by including group, time, and their interaction term. The association between age acceleration and ADI in PwMS was analyzed cross-sectionally using linear regression within the JHU cohort, with additional analyses assessing effect modification by race. Analyses were performed using R version 4.4.1, with a significance threshold of P <0.05.

### Data Availability

The study’s anonymized data will be available from the corresponding author upon reasonable request.

### Ethics statement

Human research approval was obtained from the Institutional Review Boards at JHU, UMMSM, and UCSF, with ethical approval and written informed consent from all participants for the use of blood samples for metabolomics analyses.

## Results

**Figure 1.**
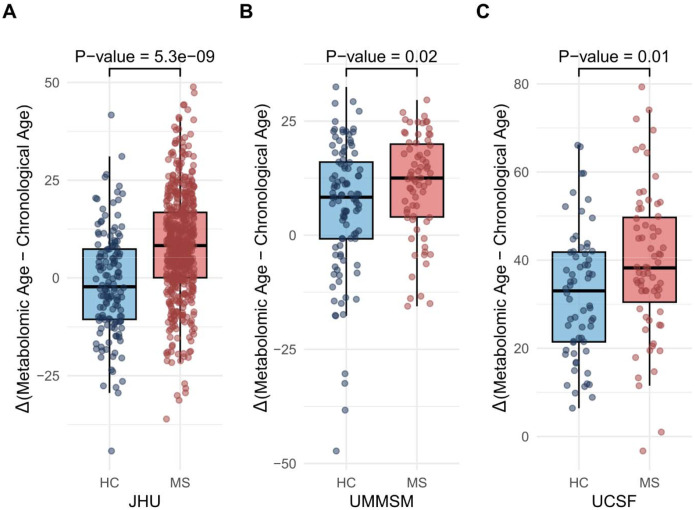
Metabolomic aging in multiple sclerosis. Comparison of metabolomic age acceleration (metabolomic age - chronological age) between individuals with Multiple Sclerosis (MS) and Healthy Controls (HC) across cohorts. **A.** Johns Hopkins MS Precision Medicine Center of Excellence (JHU) cohort **B.** University of Miami Miller School of Medicine (UMMSM) cohort **C.** University of California, San Francisco (UCSF) cohort

The JHU, UMMSM, and UCSF cohorts comprised 683, 172, and 131 samples, respectively. Detailed participant characteristics are presented in Table 1. Cross-sectionally, adult PwMS had higher age acceleration than HCs: 9.77 years in JHU (95% CI:6.57–12.97; p=5.3e-09) and 4.90 years in UMMSM (95% CI:0.85–9.01; p=0.02). POMS/CIS participants showed 6.98 years greater age acceleration than HCs (95% CI:1.58–12.39; p=0.01) (Figure 1). Longitudinal analysis of adult participants (mean follow-up: 4.57 years) revealed a faster biological aging rate in PwMS than HCs, with an average increase of 1.19 years of mAge per chronological year (95% CI:0.18, 2.20; p=0.02).

**Figure 2.**
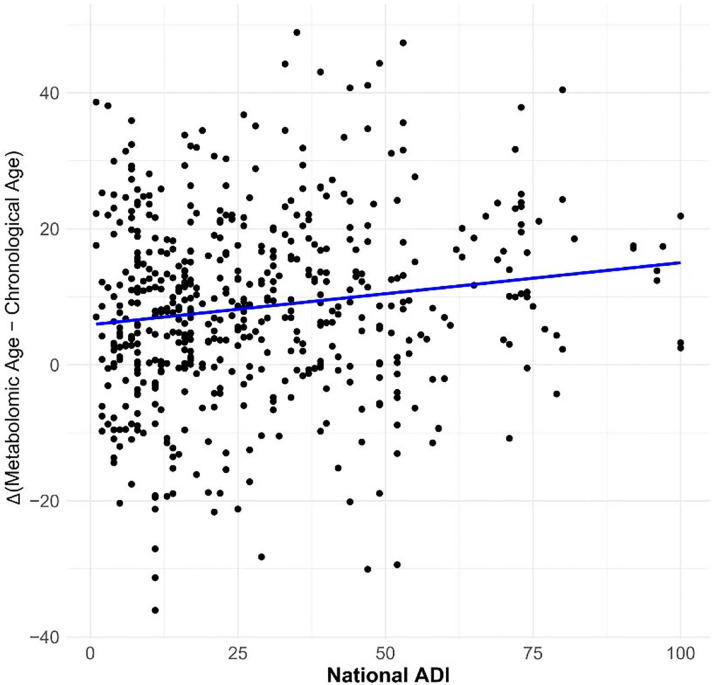
Relationship between metabolomic aging and social determinants of health in multiple sclerosis. Association of social deprivation with metabolomic age acceleration in people with MS in the JHU cohort

ADI ranged from 1 to 100 with a mean (SD) of 27.1 (20.7). In PwMS, a 10-percentile increase in ADI was associated with a 0.63-year (95% CI:0.10, 1.18; p=0.02) increase in age acceleration after accounting for cAge, gender, race, MS subtype, and MS therapy (Figure 2). We did not note effect modification by race (p=0.88).

## Discussion

We demonstrated accelerated metabolomic aging, both cross-sectionally and longitudinally in PwMS compared to HCs, aligning with previous research using other bAge markers^6,12^. Age acceleration was evident even in pediatric-onset MS and in DMT naïve patients early in their disease course, suggesting it is a core feature of MS, independent of disease duration or DMT exposure. We also demonstrated that greater social deprivation was associated with greater biological aging in PwMS.

Chronic inflammation, immune dysregulation, and cellular stress in MS likely impact aging-related processes, including mitochondrial dysfunction and DNA damage, contributing to the higher age-related comorbidities, such as cardiovascular disease and cognitive decline, further exacerbated by social deprivation^1,13–15^. The ADI reflects aspects of deprivation that contribute to chronic stress, limited health resources, and disrupted dietary patterns, expediting biological aging. Lower SES is associated with worse MS outcomes, and we provide biological insight into this relationship^2,3^. This aligns with research in cardiovascular disease, where social factors influence outcomes via aging acceleration^4^. These observations underscore the importance of addressing social deprivation in MS and suggest that mAge acceleration could be a measurable target for interventions to reduce health disparities.

This study has certain limitations. Due to the retrospective design and secondary nature of the analysis, the metabolite panels were incomplete relative to the mAge model, preventing direct mAge comparisons across cohorts. To maintain consistency, the same metabolomic clock was used for the pediatric cohort, despite it being developed in adults. Longitudinal and ADI data were available only for the JHU cohort, limiting the generalizability of ADI-associated and longitudinal findings in other cohorts. The ADI does not comprehensively capture all SDoH that may influence biological aging.

## Conclusion

We demonstrated accelerated biological aging in PwMS and its association with social disadvantage. Future studies with larger cohorts and broader SDoH assessments will help further validate these findings and unravel specific mediators of accelerated aging.

## Figures and Tables

**Table 1.** Characteristics of included study participants

Variable	JHU^1^		UMMSM^2^		UCSF^3^	
	HC	MS	HC	MS	HC	MS
No. of samples	142	541	101	71	64	67
No. of participants	66	323	101	71	64	67
No. of samples per participant (mean)	2.15	1.67	1.00	1.00	1.00	1.00
Follow-up time, mean (SD)	2.38 (2.04)	4.76 (2.74)	-	-	-	-
Age, y, mean (SD)	42.54 (12.83)	45.35 (11.90)	47.26 (16.36)	49.61 (13.21)	14.30 (2.52)	14.20 (2.72)
Age range (min, max)	22, 84	18, 72	19, 76	18, 62	8, 18	7, 18
Female gender, n (%)	106 (74.65)	390 (72.09)	74 (73.27)	55 (77.46)	30 (46.87)	32 (47.76)
Race						
Black or African American, n (%)	21 (14.79)	70 (12.94)	0 (0.00)	0 (0.00)	3 (4.69)	5 (7.46)
White, n (%)	105 (73.94)	455 (84.10)	101 (100)	71 (100)	56 (87.50)	56 (83.58)
Others, n (%)	16 (11.27)	16 (2.96)	0 (0.00)	0 (0.00)	5 (7.81)	6 (8.96)
Progressive MS, n (%)	-	152 (28.10)	-	0 (0.00)	-	-
DMT Status^3^						
Unknown	-	141 (26.06)	-	-	-	-
No DMT, n (%)	-	-	-	71 (100)	-	-
Mild efficacy n (%)	-	178 (32.90)	-	0 (0.00)	-	-
Moderate efficacy, n (%)		54 (9.98)		0 (0.00)		
High efficacy, n (%)		168 (31.05)		0 (0.00)		

1. JHU: Johns Hopkins MS Precision Medicine Center of Excellence

2. UMMSM: University of Miami Miller School of Medicine

3. UCSF: University of California, San Francisco

4. Disease-modifying therapy (DMT) status reflects the treatment participants were receiving at the time of the blood draw for the JHU and UMMSM cohorts, classified as high-efficacy (natalizumab, ocrelizumab, rituximab, daclizumab), moderate-efficacy (fingolimod, dimethyl fumarate), mild-efficacy (glatiramer acetate, interferon beta, azathioprine, teriflunomide, mycophenolate mofetil), or not on DMT.
